# Essays on Medical Statistics and Medical Jurisprudence, Adapted for the Use of Jurists and Medical Practitioners

**Published:** 1847-07

**Authors:** 


### Art. VI.

*Denkwürdigkeiten zur medicinischen Statistik und Staatsarzneikunde für Criminalisten und Aertze.* Von Dr. J. L. Casper, Professor an der Friedrich-Wilhelm's Universität., &c. &c.—*Berlin,* 1846.

*Essays on Medical Statistics and Medical Jurisprudence, adapted for the use of Jurists and Medical Practitioners.* By Dr. J. L. Casper, Professor in the University of Berlin, &c. &c.—*Berlin,* 1846. Imp. 8vo, pp. 397.

This is a remarkable work from the pen of a man who, by his extensive and elaborate contributions to medical literature, has long since deservedly acquired a European reputation. We have here *in extenso* the philosophy of medicine, dissertations upon subjects of vast importance to man in a social condition,—requiring enlarged experience, immense research, and a profound knowledge of physics and ethics for their proper elucidation. We know no one so well fitted as Professor Casper to undertake a work of this kind, involving as it does immense labour and requiring a mind well disciplined by the experience of years devoted to the study of medical philosophy in its higher and more recondite ramifications: and although some may be disposed to differ from the author in his conclusions, and to dispute the practical utility of his researches, there is no one, we think, who will refuse to admit that he deals with his subject fairly, candidly, and with an earnest desire to improve medical science and practice.

This treatise is divided into several sections, containing memoirs or essays upon subjects closely related to medical statistics and legal medicine. Some of these have already appeared in a more concise form, as monographs or contributions to the periodicals of the day:—they are here collected, arranged, and condensed; and those additions have been made which the recent progress of medical science had rendered necessary.

The first Essay is on the *Influence of the Weather on Health and Life*:—the second refers to *Experiments and Observations on the Mark produced by the Cord in Strangulation; and on the Signs of Death by Hanging*:—the third, on the *Geography of Crimes*:—the fourth, on the *Biography of a Fixed Idea*:—the fifth, on the *Mortality of the Prussian Army*: the sixth, on the *Influence of the Periods of the Day on Births and Deaths*:—and the seventh and last, on the *Phantom of a so-called Pyromania.*

The reader will not fail to be struck with the singularity of the titles of these essays, as well as with the novel and remarkable views which, when properly treated, they must necessarily lay open. To the English reader the titles will present a certain quaintness of which, without altering considerably the meaning of the author, they can hardly be deprived. We shall, however, endeavour hereafter to make good, by a copious analysis of the author's facts, any defect which may arise from an insufficient designation of the subjects of the essays.

From the preface to this work we learn that the facts here accumulated by Dr. Casper, and upon which his conclusions are based, have been collected by years of unwearied labour, while he was engaged in duties which enabled him to have free access to official, and therefore authentic documents. He candidly admits that the problems which he has here attempted to solve, are so wide in extent, that his views must necessarily be in many respects imperfect. This remark particularly applies to the Essays on the "Influence of the weather on health;"—of the "Periods of the day on births and deaths," and the "Geography of crimes." The author's conclusions are here based on statistics, and if they cannot be received as perfectly satisfactory, he will be quite contented with the admission that he has at least succeeded in pointing out the way for future inquirers. In respect to pyromania, he entirely denies its existence as a ground of irresponsibility in charges of incendiarism, thus differing from nearly all modern writers on the subject of insanity. We have considered it only just to Dr. Casper to put these preliminary remarks on record, in order that our readers may see how little he is disposed to dogmatise, or to claim infallibility for his opinions.

### I. ON THE INFLUENCE OF THE WEATHER ON HEALTH AND LIFE.

*Influence of Season.* While all are ready to admit that Health and Life are materially affected by changes in the weather, the most vague and erroneous ideas prevail even among professional men respecting the kind of influence exercised. So far back as the time of Hippocrates, it was well known that the mortality from disease varied materially at different seasons of the year. In his third aphorism this ancient observer remarks, that Spring is the most healthy, "*Saluberrimum et minimè exitiale,*" and Autumn the most unhealthy season, (*per autumnum* morbi acutissimi et exitiosissimi omnino.") Celsus adopted the view of Hippocrates. In modern times, by the use of instruments, and by the aid of tables accurately kept, many eminent statisticians have attempted to determine the degree and kind of influence which the seasons exercise upon health. Dr. Casper refers particularly to the researches of Villermé, Milne Edwards, Quetelet, Buek, and other well known statistical writers. But their researches are exceedingly defective: thus Quetelet's work refers only to the numerical deaths in a population,—the diseases which affect the living are entirely overlooked! Buek's observations are too limited to allow of any satisfactory inferences. Among the numerous difficulties which interfere with the settlement of a question of this kind, is the fact that habits and modes of life vary to such an infinite extent in different countries, that the conclusions drawn even from large numbers of instances, can have only a very limited application. Other circumstances, even in a single nation, must materially affect the results. The health of a community is liable to be affected by the temperature, elasticity, and hygrometric state of the atmosphere; and how widely different are these conditions in a crowded city and in the open country, among pauper and wealthy populations, and in manufacturing and agricultural districts!

It by no means follows that the facts which are required for the solution of this problem, can be deduced from the reports of medical practitioners in general; although this is the principle upon which tables of health and mortality are commonly founded. How many diseases (observes our author) attack individuals and disappear without medical aid being called for! What errors would have been committed in reference to the sanitary state of Berlin during the prevalence of the two influenza-epidemics, if this had been determined by reference to that portion of the population which sought medical advice, compared with the immense number of individuals who received no medical attendance whatever!

As an important and satisfactory basis for calculations of this kind, our author refers to the official reports of hospitals and of infirmaries for the poor, where these are situated amidst large populations. The influence of the weather and seasons on health can here be appreciated on an extended scale. Dr. Casper presents us with the results of observations made on upwards of 155,000 recent cases of disease occurring in public medical practice among the poor of Berlin, including the receptions into the Charity Hospital. These observations extend over a period of seven years (1833-9):—the cases for each month and season being separately numbered so as to allow of a fair comparison. He says, and we believe with perfect truth, that no table so extensive as this has ever before been published on such a subject. For the table we must refer to the original work. We can here give only a summary of the results obtained. The numbers of sick paupers, including those received into the principal hospital of Berlin in the different seasons of the seven years, were respectively in—

| | |
|---|---|
| Summer | 40,700 |
| Winter | 39,024 |
| Autumn | 37,865 |
| Spring | 37,748 |
| Total | 155,337 |

From this table we learn that in Berlin the greatest number of cases of disease occur in the summer, and the smallest number in winter. Spring and autumn are nearly equally balanced. If, therefore, in speaking of the unhealthiness of seasons, we mean to imply those periods in which *sickness is most prevalent*, it is obvious from this table, that the summer must be regarded as the most unhealthy. These observations, it is true, are confined to Berlin; but it is by no means improbable, as Casper observes, that the results would be found to be similar, not only in North Germany, but in Central Europe generally. If, on the other hand, the unhealthiness of seasons is to be measured by the relative amount of mortality in a population, then the result, as we shall presently see, will be entirely different; for under these circumstances the summer is, beyond all question, the most healthy. The difference which is here pointed out by our author is of some practical importance. The only rule for estimating the state of public health to which we have access in this country, is the rate of mortality as indicated by the quarterly and yearly returns of the Registrar-General; but it would appear, from the extensive observations collected by Casper, that when there are the fewest deaths, there is the greatest amount of illness in a given population—a result for which most of our medical readers would probably be little prepared.

Dr. Casper refers to the observations of Cless, at Stuttgardt, as confirmatory of the conclusions which he has drawn respecting the influence of the seasons on health. Cless's observations extend over a period of ten years (1828-37), and they prove incontestably that the cases of sickness are most numerous during the summer season, while they are least numerous in autumn. Local position must of course, to a certain extent, modify the results:—thus there may be in and around certain towns and cities, endemic causes, the influence of which will necessarily increase the autumnal or vernal cases of sickness.

When we come to test the relative salubrity of the different months of the year by the Berlin observations, we have, according to Casper, the following numbers representing the relative cases of sickness in each month based upon a total of 1000:

| | | | |
|---|---|---|---|
| December | 75·6* | June | 83·0 |
| May | 77·8 | September | 83·1 |
| October | 80·0 | April | 83·3 |
| November | 80·5 | July | 85·8 |
| March | 81·1 | August | 93·0 |
| February | 82·2 | January | 93·3† |

* Minimum number of Cases. † Maximum number of cases.

Hence it will be seen that the least amount of sickness occurs in December, and the greatest amount in January; the most unhealthy, on this mode of computation, following the most healthy month.

The influence of *season* on public *health* may, however, be more accurately determined by the relative amount of mortality in a given population. There can be no question, that whenever the number of deaths increases, the season must be most unfavorable to health. Another test has been proposed, which, however, is far from satisfactory, and is accordingly rejected by Casper: we allude to that based on the comparison of the births with the deaths. When the births are numerous in proportion to the deaths, this has been assumed to be indicative of a healthy season; but this is a most fallacious criterion, as the proportional numbers are liable to very great fluctuations. The registration of *deaths* appears to be carried on in a very perfect manner at Berlin. Every death is not only verified by a competent medical man, but he is required to fill up, in an official paper, fourteen different forms of inquiry respecting the deceased. These public documents therefore constitute an important basis for calculating the rate of mortality. The author admits, that in reference to pathological inquiries regarding the cause of death, they may be occasionally defective; but in reference to the present subject, the date of the death and the age of the deceased are obtained with sufficient accuracy to allow of a fair application of the results to the determination of the comparative effect of season on mortality.

These mortality tables for Berlin, compiled from official documents, include the deaths of the whole population of that city, as well as of the patients admitted into the Charity Hospital. They extend over a like period of seven years, (1833-9) and comprise 55,609 deaths, classified according to age and the month in which the death occurred. To transfer this most elaborate table to our pages is absolutely impossible. We can only give a summary of the deaths in the months,—the ages being subdivided into twenty-four periods, from 1 to 6 months to from 90 to 100 years.

| | | | |
|---|---|---|---|
| January | 4922 | July | 4670 |
| February | 4196 | August | 6020 max. |
| March | 4143 | September | 5633 |
| April | 4725 | October | 4608 |
| May | 4338 | November | 4047 min. |
| June | 4135 | December | 4172 |

An extensive table of this kind would convey no useful information, unless it were accompanied by one which indicated for each month of each year, the respective number of deaths as well as the state of the thermometer, barometer and hygrometer. By the assistance of Professor Müller, Dr. Casper was enabled to construct a weather-table of this description, based on numerous and accurate meteorological observations.

After all, the practical value of these researches must depend on the means which we possess of instituting comparisons with similar tables of health and mortality, drawn up in different countries and cities, upon like accurate principles. The official documents of the French government, on the sanitary state of Paris, published triennially, have been thus employed by Casper, having been reduced to a scale which allows of a ready comparison with the Berlin observations. The Parisian table, which he has inserted in an appendix to his work, comprises 96 months, extending from 1819 to 1826, and includes 189,196 deaths. He has also made use of Emerson's Statistics of Philadelphia, in order to place observations made in this city in comparison with those obtained from the registers of Berlin and Paris. These last comprise 120 months, extending from 1811 to 1820, and include 23,456 deaths.

The following condensed table represents the proportional mortality in different seasons in these three cities compared with the mean temperature. The total deaths were 268,261, but Dr. Casper has reduced these in the table to a standard of 100,000, and has converted the Celsius's and Fahrenheit's to the Reaumur's scale. For the convenience of our readers we have, however, again brought his temperatures to the scale of Fahrenheit, the only modification which we have thought it necessary to make in this interesting table.

| | Winter. | | Spring. | | Summer. | | Autumn. | |
|---|---|---|---|---|---|---|---|---|
| | Mean Temp. | Deaths. | Mean Temp. | Deaths. | Mean Temp. | Deaths. | Mean Temp. | Deaths. |
| Berlin . . . | 38°·5 | 24871 | 47° | 24714 | 65·3 | 26312 max. | 49°·5 | 24102 min. |
| Paris . . . | 38° | 25299 | 51° | 28630 max. | 65° | 23449 | 53° | 22619 min. |
| Philadelphia | 33° | 21158 min. | 49°·3 | 23755 | 73° | 31194 max. | 55° | 23891 |

An examination of these results will at once prove that there is no common law of Nature ruling the sanitary influence of the seasons, such as that which was supposed to exist by Hippocrates and Celsus. In not one of these three great cities, is there any similarity in the rate of mortality for different seasons. It will be perceived that the maximum mortality occurred in Berlin and Philadelphia during the *summer*, while in Paris the maximum was in *spring;* then, with regard to the minimum mortality in Berlin and Paris, this occurred during *autumn*,—in Philadelphia during *winter*. Hence, it is impossible to admit the existence of any fixed law with regard to the effect of season on mortality in different populations. As we might have inferred *à priori*, local differences, depending probably on soil, water, altitude, and the other conditions which give rise to a variety of climate, affect the results; and thus it happens that the season for the maximum of mortality in one place, is precisely that in which the minimum exists in another place.

Dr. Casper next proceeds to contrast the mortality of the present with that of the last century in reference to the influence of season. The comparative tables extend over a period of 150 years, and comprise no less than three millions of deaths. They are derived from an analysis of the mortuary registers of eighteen of the principal cities and towns of Europe. We can here only give a summary of the results, which are as follows:—the maximum and minimum rates of mortality occurred during the above period.

| | Maximum. | Minimum. |
|---|---|---|
| Winter . . . . | 8 times | 3 times |
| Spring . . . . . | 12 | 1 |
| Summer . . . . | 3 | 12 |
| Autumn . . . . . | 1 | 8 |

When the mortality of the eighteenth century is contrasted with that of the nineteenth century we have the following results:

| | 18th Century. | | 19th Century. | |
|---|---|---|---|---|
| | max. | min. | max. | min. |
| Winter . . . | 4 | 2 | 4 | 1 |
| Spring . . . . | 8 | 1 | 4 | 0 |
| Summer . . . | 1 | 7 | 2 | 5 |
| Autumn . . . . | 1 | 4 | 0 | 4 |

From these observations it is clear that, judging from the amount of mortality in a population, the *spring is the most dangerous, and summer is the most favourable season to health.* This conclusion applies equally to observations made on the periodical rate of mortality in the past and present centuries.

It was remarked by Quetelet and Villermé that the maxima and minima, at different periods, varied for the same place. In the whole of the extensive tables, collected by our author, there is, however, only one fact in favour of this view. Thus, with regard to Berlin, the maximum mortality in the eighteenth century occurred during spring, while in the present century it occurs during summer. In the former period the minimum was in winter; it is now in autumn. Villermé endeavoured to explain this by supposing that the deaths did not undergo any marked increase in the most favourable seasons; but that there was an actual decrease of mortality in those seasons, among which the maxima had hitherto fallen. This explanation, however, does not appear satisfactory. We agree with Casper in thinking that, so long as epidemics prevail with variable degrees of mortality at different periods of the year, there must necessarily be great differences in the mortality of the seasons. Some epidemics are peculiar to winter and autumn, others to summer and spring; the shifting of the maximum period of mortality, therefore, is probably due to an influence of this kind exerted unequally over a cycle of years.

*Influence of temperature.* The influence of season is, however, only one element of this important branch of statistical inquiry. Meteorological observations are required in order to show the precise influence of the weather upon health and life. Among these there is, perhaps, no condition of the atmosphere so important as *temperature.* The facts already collected in relation to the number of deaths at observed temperatures, bring us to the following conclusion.—*Extremes of temperature, whether high or low, are eminently destructive to life.* This conclusion applies equally to the four seasons of the year, the mean temperature of each season being, of course, made the standard of comparison. Thus the mean temperature of autumn at Berlin being 49°5 if the thermometer rise to 59°; this must be regarded as a high extreme with respect to that season, although it would be low with respect to summer. It is indeed a well known fact that the extremes of temperature, which occur in different seasons, are exceedingly unfavorable to health, because the human body, which becomes only gradually accustomed to change of season, is very sensitive to sudden changes from heat to cold, or *vice versâ.* We consider it unnecessary to follow Dr. Casper through the minute details into which he enters in proof of this theorem. It appears that, taking 100 deaths in the seven years already referred to in the Berlin table, there were 10·6 for the hottest summer months, while there were only 9·2 in those summers in which the temperature was lower. On the same scale the deaths were for the same period (out of a hundred) 7·5 for the hot months of spring and autumn, but only 6 for these seasons when the temperature was lower. The same fact has been noticed in other localities. Thus, in twelve summer months in Paris, having a mean temperature above 66°, there were 22,471 deaths; while in twelve summer months, of which the mean temperature was below 66°, the deaths were 21,895. Buek remarked that, at Hamburgh, under a mean temperature of from 54° to 66°, the deaths were 8·1; while, with a temperature above 66°, they were 10·1. The same observation was made by Meyer in his table of 19,251 deaths at Dresden. Here as elsewhere the maximum mortality occurred while the thermometer ranged from 86° to 92°. This agreement of results, obtained in widely different localities, proves that the increased mortality cannot, under these circumstances, be ascribed to mere coincidence, but must be a consequence of the high temperature. This inference is supported by the fact that a similar result is observed with the opposite extreme, i. e. of cold. If we refer to the Berlin table, and compare fourteen very cold months, in which the mean temperature was below 34°, with fourteen cold months, in which the temperature was milder, i. e. above 34°, it will be found that the mortality in the latter case was much lower than in the former. The respective numbers were as 8064 to 9248, or as 15·1 to 17·3 per cent. of the total mortality of Berlin. The Parisian table of mortality leads to the same result. Comparing sixteen cold months with a mean temperature below 41° with sixteen other months, in which the mean temperature was above this degree, the deaths were respectively as 32,784 to 29,024, or as a remarkable coincidence with the Berlin table precisely 17·3 to 15·3 per cent. of the total mortality of Paris. The researches of Meyer and Buek also support the view. From Buek's observations, in Hamburgh, it appears that with a temperature of

| | |
|---|---|
| 5°, the daily deaths per cent. were | 12·3 |
| 23° | 11·5 |
| 23° to 32° | 10·7 |
| 32° to 43° | 9·3 |
| 43° to 54° | 8·8 |

From these facts it is impossible to arrive at any other conclusion than that extremes of temperature, i. e. excessive heat and intense cold are destructive to life. As in regard to all atmospheric influences on life and health, this effect of temperature may be of a twofold character. On the one hand, a fact in accordance with daily medical experience, intense heat and cold may give rise to attacks of acute disease of a speedily fatal character whereby the mortality is increased. On the other hand, these influences may aggravate chronic diseases of long standing, and thereby accelerate death, thus adding to the mortality, when a more lenient temperature would probably have led to a protraction of the fatal result. A high degree of heat or a low degree of cold operates equally in the last-mentioned way; but, as it will be subsequently proved, their operation is very unequal upon individuals of different ages.

The results at which Casper has arrived, with respect to the influence of extremes of temperature on health and life, are such as might have been anticipated. Loose ideas on this subject have, for a long time, prevailed in the profession; but here, for the first time, we meet not merely with a demonstration of the fact, but a mode of determining the amount of influence, from an examination of the extensive tables which he has collected and analysed. The results here given are singularly confirmed by the tables of mortality for this metropolis, weekly issued by the Registrar-general. The effect of intense heat was strikingly shown by the prevalence and mortality attending diarrhœa during the last summer (1846.) For several weeks, during the prevalence of an unusually high temperature, the deaths from this cause among old and young were very numerous; nor, until the thermometer began to fall towards the latter end of August and the beginning of September, did the mortality sink to the average of the season. Other diseases were at the same time aggravated in their characters, and destroyed a greater number of persons than usual.* With respect to the effect of cold we cannot do better than refer to the remarks of the Registrar-general in the return for the winter quarter of 1845.

*Influence of pressure.* Since the discovery of the pressure of the atmosphere by Torricelli, more than two hundred years ago, it has been pretty generally admitted that variations of the barometer exert an appreciable, and in some instances an important influence on health and life. In spite of the large number of barometrical registers kept in all quarters with great accuracy and regularity, it is, however, not a little singular, as Casper remarks, that no attempt has ever been fairly made to apply these changes in any definitive way to the laws which govern mortality. It has been said, that with a high barometer, attacks of apoplexy are more frequent and more rapidly fatal, and that frequent fluctuations of the mercurial column are injurious to health; but even then common opinions rest upon no well observed data: their correctness yet remains to be tested by facts.

* The summer of 1846 was remarkable for a long continued high temperature. In the week ending August 8, when the mean temperature was 5° above the mean of this the hottest month in the year, the deaths were no less than 237 above the summer average! The same increase of mortality was observed in New York. We extract the following from a medical periodical: "During the hot weather, in one week, in New York, the extraordinary number of 4025 deaths occurred, or more than one in every 1000 inhabitants. Of these, 31 were caused by apoplexy, 21 by sun-stroke, and 52 by cholera infantum. 169 were of children under one year; of married women only 58 died."

According to our author, a high or a low degree of pressure has a more striking and more uniform relation to the rate of mortality, than even a high or low temperature, and the position which he undertakes to establish is this :—*In nearly all the seasons of the year a high atmospheric pressure increases, and a low pressure diminishes the rate of mortality.*

We consider it unnecessary to reprint here the very copious table of barometrical observations for each month of seven years (1833-9), four only being excepted, (Aug. to Nov., 1837), during which the cholera prevailed in Berlin and materially affected the general rate of mortality. To each month is attached the mean pressure compared with an assumed standard, and in another column the number of deaths. According to Casper, the mean pressure at Berlin is 336,000‴,* or 29·83 inches English. In order to divide the 80 months into two equal parts, the standard height, as mean pressure, is assumed to be 336,361‴, or 29·86 inches English, that is, rather lower than the English standard, which is commonly taken at 30, although this number is probably taken rather from convenience than from actual observation.

In forty months with the barometer above 29·86 inches, there died in Berlin 25,221 persons; and in forty months with the barometer below this point, there died 25,021. To give a more correct idea of the proportional rate of mortality, it may be stated that under a high barometer, the monthly deaths were 630·5, (or 18·48 daily); while under a low barometer, the monthly deaths were 625·5, (or only 18·25 daily.) For every 100 who died while the barometer was low, 101·2 died, when it was high.

The destructive influence of a high pressure will become more apparent, if we compare the mortality in the weeks in which there was an extreme high, with that of the weeks in which there was an extreme low pressure. Thus, in thirteen months in which the mean pressure was 338,000‴, (29·98 inches E.), the deaths were 8400, or an average of 646 per month, or 21·54 daily. On the other hand, in the thirteen months during which the average height of the barometer did not exceed 334,800‴, (29·72 inches E.), and was for the most part below this point, the mortality was only 621 per month, or 20·79 daily;—or for every 100 persons who died in the latter case, 103·9 died in the former, a difference sufficiently well marked in favour of a low atmospheric pressure. It becomes an interesting subject to consider whether this influence of pressure is equal at all seasons of the year. Casper's researches show, that so far as it relates to Berlin, there is a variation with the season. We must here again content ourselves with a summary of his results:

| High Barometer. | Av. deaths per month. | Low Barometer. | Av. deaths per month. |
|---|---|---|---|
| In 11 winter months . . | 656 | In 10 winter months . | 607; — 49 |
| 8 spring „ . . | 586 | 14 spring „ . | 679; + 93 |
| 14 summer „ . . | 627 | 5 summer „ . | 619; — 8 |
| 7 autumn „ . . | 649 | 11 autumn „ . | 576; — 73 |

* The author adopts the highly inconvenient practice of reckoning the barometrical heights in lines. A line is equal to ·0888 inch. This, being a circulating decimal, can have no strictly equivalent representative number in fractions. A line is commonly said to be equal to 1-12th of an inch; but this does not quite express the measurement, for a line is rather more than this in length. 336 lines as above would be equal to (·0888 × 336) 29·836 inches English, the mean pressure for Berlin.

Or, to give the results in a more definite form, the deaths were:

| | High Bar. | | Low Bar. |
|---|---|---|---|
| In Winter . . . . | 108·0 | to | 100 |
| Spring . . . . | 86·3 | to | 100 |
| Summer . . . . | 101·3 | to | 100 |
| Autumn . . . . | 112·6 | to | 100 |

These differences are not merely local;—the same results with slight modifications have been met with in other places, and they all point to one general conclusion, which has been hitherto scarcely noticed, namely that *the influence of atmospheric pressure upon human life is not uniform at all seasons of the year.* That this result, derived from the Berlin tables, is not a mere coincidence, or dependent upon a small number of observations, will be presently made evident by a reference to the Parisian tables. In the mean time, it would be interesting to inquire what are the diseases either of a new or chronic character which add so materially to the mortality under a high barometer during the autumnal season, as well as those which on the other hand have their mortality diminished under a high barometer in spring; but facts are wanting to enable us to solve these questions. In the springs of these years, catarrhal and rheumatic diseases, with and without fever, cholera, hemorrhages, and congestive diseases were observed to prevail. The result proves that the conclusion that the prognosis for such diseases is more favorable under a low atmospheric pressure, is decidedly premature. In Paris, it was remarked that the mortality from disease was diminished during autumn under a moderately high barometrical column. Thus the following were the results with a pressure *above* 755·96 mm. (29·7 inches E.), and with a pressure below this point:

| | Bar. above 29·7. | | Bar. below 29·7. |
|---|---|---|---|
| In 16 winter months . . | 2021 | In 7 . . . | 1971 — 50 |
| 11 spring . . . | 2334 | 13 . . . | 2129 —142 |
| 13 summer . . . | 1841 | 9 . . | 1831 — 10 |
| 8 autumn . . . | 1754 | 15 . . . | 1804 + 50 |

Or,

| | High Bar. | | Low Bar. |
|---|---|---|---|
| In Winter . . . . | 102·5 | to | 100 |
| Spring . . . . | 106·4 | to | 100 |
| Summer . . . . | 100·5 | to | 100 |
| Autumn . . . . | 97·2 | to | 100 |

With the exception of this slight difference according to the seasons, the prejudicial influence to health of a high barometrical pressure, is as strikingly and as uniformly shown by the Parisian tables as by those of Berlin. We state the facts, but must pass over the series of observations. The Parisian tables prove that, as in Berlin, the deaths under a high, were to those under a low barometric pressure as 101·3 to 100; or as a daily ratio 66·32 to 65·42. A comparison of the extremes establishes with equal clearness that there is a manifest difference on the side of a low barometrical pressure in favour of the health of a population. In twelve months, during which the barometer was moderately high, the monthly deaths in Paris were 2016, and the daily deaths 67·20. In twelve other months, when the barometer was somewhat lower, the monthly deaths were 1985 and the daily deaths 66·17, the ratio in the two periods being 101·5 : 100. The observations made by Meyer, in Dresden, equally lead to the conclusion that the mean rate of mortality is greater under a high, than under a low barometrical pressure.

It would be interesting to connect, if possible, the diffusion and mortality of epidemic diseases with the variable states of atmospheric pressure; but our knowledge of the causes and essence of these diseases is so limited, as to render it impossible at present to say whether any such influence on their progress does or does not exist. During the prevalence of epidemic cholera in Berlin, extensive observations were made by Casper on the barometer; and the changes in this instrument were compared with the daily fluctuations in the rate of mortality; but nothing certain could be deduced from them. Dr. Prout observed a remarkable change in the state of the atmosphere about the period at which this disease broke out in London. This gentleman states that for more than six weeks previously to the appearance of the cholera in London, he had been daily engaged in performing experiments to determine with accuracy the weight of a given quantity of air. On a particular day, the 9th of February, 1832, the weight of the air suddenly appeared to rise above the usual standard. The rise was at first considered to be due to some accidental error, but a careful repetition of the observations proved that this was not the case. Dr. Prout supposes that the increased weight of the air in London at this time, depended on the diffusion through the lower strata of some heavy gaseous body. About the 9th of February, the wind which had previously been west, veered round to the east, and remained chiefly in that quarter till the end of the month. It was precisely at this time that the first cases of epidemic cholera were reported to have occurred in London, and from that date the disease continued to spread.*

There can be no doubt that the cholera follows certain laws peculiar to itself, a fact which appears to be established by the mode in which its progress and mortality were influenced throughout Europe by local circumstances. Other epidemics of a less violent kind are, however, doubtless, influenced by the meteorological conditions of the atmosphere; but exact observations are wanting to show to what extent this influence operates.

*Influence of the hygrometric condition of the atmosphere.* In spite of the popular belief to the contrary, Dr. Casper contends that a humid state of the atmosphere is more healthy, and attended with a lower rate of mortality than a dry condition of this medium. The difference, he thinks, is easily explicable by the different nature of the diseases which are liable to be caused by these opposite hygrometric conditions of the air. When there is a surplus of humidity,—catarrhal, rheumatic, and glandular (scrofulous) diseases, with intermittent fevers, prevail. In a given population, there may be therefore much sickness, but the rate of mortality may either not be increased, or only in the very smallest degree. The result is far otherwise with those diseases which are apt to take their origin in a dry state of the atmosphere, as inflammations and hemorrhages, including apoplexy, with their serious sequelæ. Experience is decidedly in favour of this distinction. Thus the Berlin and Parisian tables, which have been already employed to show the influence of pressure, will serve to prove this remarkable influence of the hygrometric condition of the air. In the periods referred to, out of 100 deaths there were—

* Chemistry, Meteorology, and the Function of Digestion, 3d edition, p. 311.

| | In Berlin. | In Paris. |
|---|---|---|
| During very dry months . . . . . . | 52 | 50·5 |
| In wet months . . . . . . | 48 | 49·5 |

This comparatively beneficial influence of a humid atmosphere appears to exist equally throughout all seasons of the year, but very wet summers are rare in Middle Europe; hence, in order to obtain a proper comparison of this with other seasons, a much more extensive series of observations would be required than those to which we have at present an opportunity of referring. Making therefore due allowance for this deficiency, the following table represents the comparative rate of mortality during dry and wet seasons in Berlin and Paris. The numbers given under the different seasons are reduced to a standard of 100 deaths:

| | Berlin. | | | | Paris. | | | |
|---|---|---|---|---|---|---|---|---|
| | Winter. | Spring. | Summer. | Autumn. | Winter. | Spring. | Summer. | Autumn. |
| Dry . . | 13·5 | 12·9 | 13·1 | 12·4 | 13·2 | 14·5 | 11·7 | 10·9 |
| Wet . . | 11·7 | 12·4 | 12·6 | 11·1 | 11·6 | 14·1 | 11·8 | 11·9 |
| Difference . | —1·8 | —0·5 | —0·5 | —1·3 | —1·6 | —0·4 | +0·1 | +1·0 |

Admitting that there exists here a difference in favour of the wet seasons, Dr. Casper thinks that the result will be made more obvious by comparing months according to temperature and humidity. The Berlin tables are in this respect imperfect, for the reason that, during the whole period, they present only three humid warm months, a number much too small to allow of the institution of a fair comparison. Our author, therefore, bases his calculations upon the Parisian table, to which the same objection does not apply. This table furnishes us with 10 months which were warm (compared with the mean temperature) and humid; 12 months warm and dry; 14 cold and humid; and 18 cold and dry. If the rate of mortality during these periods be compared with the average mortality for the respective months (as for example, the mortality of a dry and cold January, with the average mortality for this month), we obtain as the result of this extensive series of calculations, the following valuable summary:

| | Mortality in the months which were | Average mortality of these months. | Proportion. |
|---|---|---|---|
| Humid and warm . . | 1853 | 1842 | 100·6 : 100 |
| Dry and warm . . | 1863 | 1829 | 101·8 : 100 |
| Humid and cold . . | 1882 | 1923 | 97·8 : 100 |
| Dry and cold . . | 2029 | 1986 | 102·1 : 100 |

We thus arrive at the conclusion *that a humid state of the atmosphere is more favorable to health and life than that which is dry;* for taking the average mortality in a given population to be 100, the deaths will be 101·9 in dry seasons, and only 99·2 in wet seasons. It is evident, not merely from these but the Berlin observations, that *no state of the atmosphere is so prejudicial to health and life as that of dry cold;* and again, contrary to the opinion of Kopp and others, it is not humid warmth, but humid cold which tends to increase the rate of mortality in a population.

From this review of the influence of the physical changes in our atmosphere upon life and death, the important practical question arises how or in what way these changes lead to the origin, prevalence, and diffusion of various forms of disease. Many difficulties are opposed to the proper solution of this question. In most tables of mortality, especially those of a public kind, the nomenclature of disease is so uncertain as to render them in this point of view useless. Dr. Casper has, therefore, judiciously confined his analysis to a few diseases, the real description of which there could be no reason to doubt. He has, however, made a further subdivision, selecting from these diseases, those only (forming a small number) the origin and mortality of which are known to be influenced by the physical states of the atmosphere. Further, the diseases have been reduced to classes in order to condense the results. The tables kept in hospitals have been taken in preference to those which were of a public kind; since the diagnosis and description are better given, and the medical officers in these institutions take more pains in keeping an accurate record of facts, than those who are officially employed by government. A valuable table of this kind including the patients received in the hospital and the infirmaries for the poor, in Dresden, has been contributed by Meyer. It extends over a period of ten years (1828-37) and comprises 27,067 cases of disease. Dr. Casper makes use of this table according to the principles already described; we can, however, do no more than advert to its leading features. In the first place, although it differs from the Berlin table in showing that the receptions per 100 during summer were, when compared with those during winter, as 22 : 27, and therefore that the maximum degree of sickness did not exist in Dresden during the summer season; yet the results clearly corroborate the inference already drawn, that the minimum mortality occurs during summer, and the maximum during spring. The respiratory organs are proved to be especially liable to dangerous attacks during spring. One third of all the cases of inflammatory diseases of the chest collected by Meyer (360 : 1092=32·9 : 100), occurred during the vernal months. The per centage of cases of disease of these organs was during summer only 18·2; but in the winter months, it rose as high as 25·7. Again, the maximum number of cases of consumption occurred in spring, 29·9 : 100; and the minimum, in summer, 21·7; and, although many of the cases received into the hospital list may really have been of some standing, the results prove that at a certain period the diseases assumed an aggravated character; a result which admits of no other explanation, than that of being dependent on atmospheric influence. The increased mortality during spring among those labouring under this disease, is a sufficient proof that this season of the year exercises an important influence over it.

Meyer's table confirms the general observation, that congestive diseases and hemorrhages are much more prevalent during winter; but the results of the influence of season upon these diseases in Dresden, are not so striking as those which have been obtained in several other places. This influence becomes more apparent when we observe the number of *deaths* which take place suddenly at this season from hemorrhages and congestions—a clear indication of the greater severity of the isolated attacks. We have here an instance of the manner in which cold operates, by driving the blood from the surface to the centre; and every practitioner knows how much more frequently death takes place from cerebral and pulmonary hemorrhage during winter, than at other seasons of the year; and during long and severe winters, than in those which are short and mild. In confirmation of this view, Casper quotes from the second report of our Registrar-general the return of 2382 deaths from diseases of a congestive or hemorrhagic tendency. It appears that these were distributed over the four seasons of the year as follows:

| Jan. — March. | April — June. | July — Sept. | Oct. — Dec. |
|---|---|---|---|
| 729 = 30·6 : 100 | 543 = 24·4 | 471 = 19·7 | 599 = 25·1. |

The table published by Ferrario, of the number of sudden deaths (through apoplexy) which took place at Milan during a period of fifty-six years (1774-1830), in which the influence of weather, season, age, and occupation on this disease is accurately traced, furnishes additional proof of the exceeding prevalence of apoplexy during the colder months of the year. Out of 10,432 deaths from this cause, we have the following numbers for the respective months:

| | | | |
|---|---|---|---|
| January | 1176 | May | 829 |
| December | 1075 | October | 822 |
| February | 1030 | September | 718 |
| November | 963 | July | 689 |
| March | 956 | June | 681 |
| April | 848 | August | 645 |
| | Total, 10,432. | | |

From this it will be seen, that the number of cases of apoplexy which occurred in January, was nearly twice as great as the number registered in August.

Meyer's table only confirms what has been long a matter of experience, that diarrhœa and other disorders of the intestinal canal are far more prevalent in summer and autumn, than at other seasons of the year. From a table prepared by Cless, and reprinted by Casper, of the patients received into the hospital at Stuttgardt, during ten years, from 1828 to 1837, diseases of this kind formed only 6·5 per cent. of the cases which occurred in winter and spring; while they constituted no less than from 40 to 47 per cent. of those admitted in summer and autumn. It is, however, obvious that many causes, independently of atmospheric influences, tend to produce these diseases. Cases of bilious fever with gastric symptoms, varied equally with the season; in winter and spring these amounted to 440, in summer and autumn to 774.

Dr. Casper is inclined to think, contrary to the general opinion, that the disposition to attacks of syphilis and gonorrhœa is influenced by season. In his opinion Meyer's table furnishes proof of the existence of an influence of this kind. It appears that of 839 cases of syphilis and 71 of gonorrhœa, there was the following distribution of attacks according to the season of the year:

| | Winter. | Spring. | Summer. | Autumn. |
|---|---|---|---|---|
| Cases of syphilis | 205 | 174 | 232 | 228 |
| „ gonorrhea | 16 | 19 | 25 | 11 |
| | 221 | 193 | 257 | 239 |

The occurrence of these cases would thus appear to be by 7 per cent. more frequent in summer than in spring. Other causes besides those depending on season may, however, have some influence in producing a greater number of these cases during summer. The author was unable to make any comparison according to season, of the admissions for these diseases into the great hospitals of London and Paris. It has frequently been observed by practitioners of long standing, that venereal cases occur more frequently at one period of the year than another; but further researches are required in order to determine the degree in which this more frequent occurrence of syphilis is to be ascribed to the effect of season.

It is of great practical interest to compare at particular seasons, the relative amount of sickness (*morbility*) with the relative amount of mortality. This has been already in part done, but it must be confessed that we are yet unprovided with any certain means of measuring the degree to which the severity or mildness of particular diseases may be influenced by season; although researches on this subject would have a most important bearing on prognosis in particular cases. The comparison of tables of *morbility* (to employ Dr. Casper's term) with those of mortality when accurately made under similar conditions, will enable us to form an approximate judgment, and to deduce some useful inferences of a practical kind.

Our author then furnishes us with a table of upwards of 52,000 deaths, occurring in Berlin during a period of ten years 1830-39, of which the causes were well ascertained. Those fatal diseases only were selected which were known to be more or less under the influence of atmospheric changes. We do not give this table, because we think a summary of the results will fully answer the purpose of developing the nature of the question which the author here proposes for solution. This table confirms the views already brought forward; thus with respect to inflammations attacking the brain, throat, lungs, and abdominal viscera, we find that out of 100 deaths from this class of diseases there were:

| In summer. | In autumn. | In winter. | In spring. |
|---|---|---|---|
| 19·0 | 23·6 | 26·6 | 30·5 |

The deaths from inflammation of the lungs and acute hydrocephalus, were so numerous during spring, that more than one third of all who laboured under these diseases perished at this season: a fact which may furnish a hint to the practitioner when forming his prognosis at the bed-side of a patient. According to Buek, the deaths in Hamburg from diseases of an inflammatory kind, had the following daily average:

| In summer. | In autumn. | In winter. | In spring. |
|---|---|---|---|
| 62 | 70 | 80 | 3 |

And he also remarked what is a striking correspondence with the Berlin observations, that one third of the deaths from inflammation of the lungs and hydrocephalus, occurred during spring. The same inference is deducible from the deaths registered in the Edinburgh infirmary for three years, from 1822-4. These facts connected with the fatality of inflammatory diseases, are derived from 9400 cases observed during the second and third decennial periods of the present century, in Berlin, Hamburg, and Edinburgh; and we may thus take it as an established fact, that of all seasons of the year, the winter has the greatest tendency to produce these diseases; while the spring appears to render them most fatal. This great fatality is especially seen during the latter season in cases of pneumonia and pleuritis.

Let us now pass to the examination of that difficult question so often raised; namely, the influence of season and climate on *phthisis pulmonalis*. Dr. Casper informs us of what we have already learned from the researches of Sir James Clark and others in relation to this disease, that it is one of the most universally prevalent and fatal; and that in every climate large numbers of persons are annually cut off by its attacks. The advance recently made in science has enabled the physician, without any great difficulty to detect the existence of tubercular disease in its earliest stages. The stethoscope, plessimeter, microscope, the scalpel aided by profound chemico-pathological researches, have furnished the most valuable materials to assist the diagnosis of the physician; but in spite of the assistance thus rendered, the art of medicine appears still to be powerless before this devastating disease. Medical statistics with respect to *phthisis* have been for a long period accurately kept in different countries; and tables of its attacks and rate of mortality are so numerous as to render the selection of satisfactory data a task of no great difficulty. From these documents we learn that medical treatment has had so little influence in arresting the progress of this scourge to mankind, that under every variety of climate, from a fourth to one sixth of all those who die from disease, are cut off by phthisis pulmonalis. From the many excellent works on this subject, in various languages, and from the tables published by different authors, Dr. Casper has made a selection of all those cases which appeared to resemble each other in their conditions, and in the circumstances which attended them. The table which he has thus constructed, refers to the period of the last twenty or thirty years. We here give it as containing the most recent and accurate statistical information, which we have on the relative degree of mortality occasioned by tubercular phthisis.

MORTALITY FROM PHTHISIS PULMONALIS.

| Places. | Periods in years. | Deaths from phthisis. | Total deaths. | Ratio of deaths from phthisis. |
|---|---|---|---|---|
| Berlin . | 10 . 1830-9 | 12800 | 73216 | 1 : 5·7 |
| Paris . . | 4 . 1816-19 | 15375 | 85339 | 1 : 5·5 |
| London . . | 2 . 1840-1 | 14562 | 91565 | 1 : 6·2 |
| Hamburg . | 3 . 1823-5 | 2360 | 10963 | 1 : 4·6 |
| Stuttgardt . | 10 . 1828-37 | 924 | 4356 | 1 : 4·7 |
| New York . | 11 . 1816-26 | 7466 | .. | 1 : 5 |
| Philadelphia . | 7 . 1820-6 | 3590 | .. | 1 : 7·7 |
| Baltimore . | 8 . 1819-26 | 2243 | .. | 1 : 6·7 |
| Boston . . | 7 . 1820-6 | 1481 | .. | 1 : 5·9 |
| | | 60801 | | 1 : 5·8 |

From this table, comprising upwards of sixty thousand deaths among different populations, we find that on the average *one death out of six* is caused by this formidable disease; in England about 59,000 lives annually fall a sacrifice to it. In comparison with this amount of mortality, the devastations occasioned by the plague or by a transitory pestilence like the Asiatic cholera, sink into nothing!

It was supposed by the ancients, and it is even now a subject of popular belief, that the autumnal season, i. e. the fall of the leaf, is the period in which consumption is most fatal; but the comparative number of patients admitted at different seasons, as well as the tables of mortality, prove beyond all doubt that spring is by far the most dangerous season to the phthisical. Observations carefully made in Vienna, Paris, Strasburg, Geneva, Stuttgardt, London, Hamburg and Berlin, all combine to show that the maximum number of phthisical patients die in the spring; and after this in winter; while the minimum deaths occur in summer and autumn. On this account, observes Casper, it is the practice with physicians in England and on the Continent, to send their patients to pass the winter in a milder climate; a fact which shows how necessary it is to extend our inquiries into the influence of climate over this malady. It is not a little singular to trace the history of those who are thus banished from their country. According to Heineken, quoted by Casper, of 35 consumptive patients who went to Madeira in 1821, 2-3ds died on board; 3 died in the first month after their arrival; 5 to 6 survived the winter, and about the same number survived the following spring. From 3 to 4 lived to the second winter, but of the whole number there were only 13 living in 1824. The graveyards of Rome, Naples, Marseilles, Pisa, Nice, and Malta, bear ample testimony to the fatality of this disease, among those who have been induced to seek a foreign clime in the vain hope of recovery!

A question of this kind, however, is deserving of still further examination; and in order to throw greater light upon the subject, our author has given in a distinct table in his appendix, a most extensive series of observations comprising the varying states of the atmosphere with the relative monthly mortality from consumption, for a period of nine years (1830-38). The facts were collected by Mädler, and they comprise no less than 11,472 deaths, a number amply sufficient for the purposes of scientific inquiry. We agree with our author, that so extensive and accurate a table as this, has never before been published; and the facts which it reveals are calculated to remove many absurd errors respecting this disease, which have been too credulously received as truths by medical practitioners.

The mean barometrical pressure for Berlin during the nine years, was 333,551‴ (29·79 inches E.), and the average number of deaths from phthisis in each month amounted to 106. In 84 months, the barometer was above this average; and in 36 of these months the deaths from phthisis were fewer, while in the remaining 48 these were in number greater than the monthly average. In 24 months, the monthly mean of the barometer was less than that above assigned, and under this low atmospheric pressure, the deaths in 14 months exceeded, and in 10 months were less than the usual monthly average. As far, therefore, as any inference can be drawn from the relative amount of mortality and the pressure of the atmosphere, it appears that proportionally fewer phthisical patients die when the barometer is high, than when it is low; a result which is the reverse of that obtained from the analysis of the general mortality of a population.

It may now be well to inquire what relation, if any, subsists between the mortality from this disease and fluctuations in temperature; for there is a general opinion, that there is some close connexion between them; Mädler's table furnishes us with the following facts: The mean temperature of the nine winters (comprising the months of December, January, and February) was 33°. The coldest winter (1838) had a mean temperature of 24°; and in that season 393 phthisical patients died; the warmest winter (1834) had a mean temperature of nearly 36°, and the number of deaths was nearly the same. The two winters of 1832 and 1835, had nearly the same mean temperature (32°·7 and 33°·5 respectively); and yet there died in the first 345, and in the last only 284 patients from this disease; a difference which is too great to be referred to a difference in the progressive increase of population. The warmest of the 8 springs

(1830) had a mean temperature of 50°; and the mortality was almost as much above the average as in the coldest (1837), in which the mean temperature was under 44°. How is it possible to deduce from conflicting results of this kind any natural law?

Although there may appear to be some relation between the mortality from phthisis and the sudden fluctuations of the barometer when extremes are taken, yet by a close analysis of the table, even this slight relation disappears. These fluctuations were at their maximum in the summer of 1830, and at their minimum in that of 1831, and there were 26 more fatal cases of phthisis in the former, than in the latter season. On the other hand, there were in the summer of 1834, 30 deaths more than in that of the following year, although both of these summers had the same mean in their barometrical fluctuations. In the summer of 1838 there were 50 more deaths than in that of 1832, and yet these summers were almost identical in their barometrical variations,—a clear proof that the fluctuations of this instrument could have had no influence on the mortality from this disease.

The facts displayed in this table show that changes of temperature are as little connected with the rate of mortality from phthisis. The greatest variations in the thermometer were observed in the winter of 1831, the smallest in that of 1832; and yet there was scarcely any difference in the deaths from phthisis during these two seasons. In the former, the deaths were 356, in the latter 345. The winters of 1836 and 1837 were very similar with respect to temperature; and yet the deaths were respectively 260 : 342. The maximum difference in the range of the thermometer was observed in the winter of 1830, while the minimum difference was in the winter of 1833; in other words, there was during this season a remarkable uniformity of temperature, and yet, notwithstanding these extreme differences, the numbers of phthisical patients who died during the two seasons were nearly equal, being 326 : 334. As to the effect of temperature in accelerating death, it appeared, therefore, to be quite immaterial whether the phthisical patients breathed and lived in an equally warm atmosphere, or whether they were exposed to frequent changes of temperature irrespective of the hygrometric condition of the air.

With respect to this last condition, i. e. the hygrometric state of the atmosphere, it appears from the table that this had really a well-marked influence on the fatality of phthisis. The facts already quoted on a large scale show that, in general, humid is more favorable to health than dry air, and from this table we find the following results were obtained. The deaths from phthisis were:

| | |
|---|---|
| In dry winters | 367 |
| In winters, partly dry and partly humid | 338 |
| In dry summers | 291 |
| In mixed summers | 289 |
| In dry autumns | 298 |
| In mixed autumns | 263 |

The springs formed an exception, for in those which were dry the average deaths were 329, while in those which were of a mixed kind they were on the other hand 363.

It is well known that there is considerable difficulty in determining the prevalence of particular *winds;* and the results obtained by Mädler, with respect to the effect of certain winds in phthisical mortality, are somewhat of a negative character. Without quoting the details, it may be sufficient to state that the mortality remained nearly uniformly the same in two similar months, when in these months the prevalent winds were in diametrically opposite quarters.

Dr. Casper has thus far endeavoured to show from accurate data, that the different states of the atmosphere and weather exert but a very feeble influence on the mortality caused by phthisis; and that the only appreciable influence which appears to exist, is that which is dependent on *season:* and here, he observes, it is necessary to bear in mind that health and life are affected by change of season in other ways than by mere differences in temperature, pressure, &c. Thus the occupations, mode of life, and food of large sections of a population, are well known to vary with the season; and this of course must have an influence upon those who are suffering from disease. Our author then raises the question as to how far, with a knowledge of these facts, we are justified in sending persons affected with phthisis into the South of Europe. It certainly is, as he remarks, rather an important fact, that we are carrying on this practice to a great extent upon merely hypothetical grounds of a supposed benefit from change of climate. We are as yet unprovided with any satisfactory statistical tables of the frequency and mortality of this disease among the populations of those countries to which we consign our own patients; and thus, can institute no comparison with those which have been drawn up in Northern Europe. In one of the most recent works on the Climate of Nice, by Ernst, this author takes a widely different view of the question from that taken by Casper; and his tables of the mortality in and around Nice, lead to a conclusion the reverse of that drawn by this statistician. Again, Dr. Richelmi, an eminent practitioner of many years' standing in that district, states that in ten years there were 6987 deaths, and only 107 of these, i. e. one in *sixty-six,* due to phthisis. Casper objects to this statement, as proving too much. A solitary result so conflicting with accurate observations made upon this disease, in all other countries, must tend to create distrust in the correctness of the data, especially as the sources of the observations and the decennial period in which they were made, are not mentioned. However, in Casper's opinion, the table itself contains intrinsic evidence that the statements are inadmissible. According to Richelmi, the general mortality in the country around Nice, is 1 : 37; in Nice itself, 1 : 32. Our author believes it to be absolutely impossible, from his knowledge of Nice and its environs, that such an enormous difference, as more than one seventh, should exist in the relative mortality of the town and country. Tables which lead to conclusions so evidently erroneous cannot be otherwise than faulty. Dr. Casper contends that there is no proof that any benefit is obtained by the phthisical in changing from a northern to a southern climate. The contrary opinion has arisen from an imperfect acquaintance with the influence exerted by the atmosphere and climate on this disease; and its existence shows the necessity for procuring accurate statistical information. As an instance of the want of any scientific principle in recommending a change of climate, he states, but without quoting names, that an eminent English physician of great practical experience, and a German *literary* (!) physician, have recently and on widely different grounds adopted the practice of advising their phthisical patients to make a journey to—RUSSIA!

*Nervous Fever.*—(*Nervenfieber, Der Darmgeschwür-typhus.*) Dr. Casper now passes to the consideration of another form of disease exceedingly prevalent, which has received as much attention from science as tubercular phthisis, but which in a therapeutical point of view is nearly as intractable. This is nervous fever. It appears that this fever, with gastro-enteritic symptoms and ulceration of the bowels, is very prevalent and fatal in Berlin; and at certain seasons of the year spreads in an epidemic form. Under these circumstances it might be supposed that there would be no great difficulty in tracing a connexion between it and atmospheric changes; but the contrary is the fact. We quite agree with the author that, in these investigations, negative results are not to be despised; for if attended with no other benefit, they have at least the effect of removing error and scientific prejudices. The following results are, therefore, worthy of being recorded.

In the 108 months of the nine years referred to, there died in Berlin, from this fever, on the average of each winter month 27,—of each spring month 18,—of each summer month 23,—and of each autumnal month 41. The influence of season over this disease is, in Caper's opinion, well marked. The results were obtained from nearly three thousand (2947) deaths, and therefore it may be fairly stated, *that this fever is most prevalent and fatal in autumn, and least prevalent and least fatal in spring.* By the closest analysis, our author was unable to deduce from the observations, any inference which showed that the rate of mortality from this disease, was at all influenced by barometrical, thermometrical, or hygrometrical changes in the atmosphere, or that the deaths were greater or less, under the prevalence of particular winds.

*Inflammatory Diseases.* The diseases under this section, including hydrocephalus, croup, laryngitis, bronchitis and pneumonia, are notoriously influenced both in respect to their origin and diffusion, as well as in their rate of mortality by atmospheric changes. Out of 1000 cases of this description, there died in the spring 322, in the winter 276, in the autumn 228, and in the summer only 172.

Various and conflicting opinions are entertained respecting the influence of barometrical fluctuations and of particular winds, such as the north and easterly winds, over this class of diseases: these have long required testing by experience and observation. Casper has compared the number of deaths which occurred from inflammatory diseases when the barometer was above and below the mean, including the sudden fluctuations of this instrument, with the rate of mortality; and he has ascertained that there is no kind of connexion or dependence of one upon the other. The rate of mortality appeared to be quite unaffected by the variations in the *pressure* of the atmosphere.

The effect of temperature appeared to be somewhat more strongly marked, although not in the way commonly supposed. Thus it was ascertained by a comparison of years, that a high temperature during spring rather tended to increase the rate of mortality from inflammatory diseases. In the four coldest springs 1832-5-7-8, there died 813 persons; while in the four warmest, 1830-1-4 and 6, there died 880 persons. This conclusion was strengthened by a comparison of the monthly deaths in March, according to whether that month was colder or hotter than the mean. The mortality at other seasons also indirectly confirms it. Thus, in the four hottest summers, the deaths were 656; in the four coldest, only 610; in the cold month of July 1832, only 53; and in the hot month of July 1834, 75. We need not enter into further details, all leading to the same result; but, making full allowance for increase of population and taking the average deaths for each year, we inevitably arrive at the conclusion, *that cold winters, warm springs, summers, and autumns, increase the amount of mortality from inflammatory disorders, and vice versâ.*

It is generally supposed that a very dry state of the atmosphere predisposes the respiratory organs to inflammation, and increases the mortality from these diseases; but facts appear to be against the correctness of this opinion. In nine wet months, the deaths from inflammation exceeded the average in four; but did not reach it in five. A similar result was obtained by taking the deaths in nine dry months. In the dry winter of 1830, 220 died; in the following winter equally dry, only 164; in that of 1836, which was very variable with respect to humidity, there were 180 deaths; and in the following equally variable winter, they were 249.

The examination of the table for the whole of the nine years, showed that the influence of the wind on the number of deaths from this cause, was inconsiderable. There were but slight variations under the prevalence of different winds; but no satisfactory inference as to their effect could be drawn from the observations.

As a general conclusion, it may be observed that the seasons of the year, taken on the whole, exert a pretty constant and universal influence on the mortality produced by disease. The special conditions of the atmosphere in relation to this influence, cannot at present be arranged under any law of nature. A greater delicacy and accuracy in our instruments of research, and a more extended series of observations, are required before this desirable object can be attained.

*Age.* Man is not the same at all periods of life; the stages of infancy, of adult and old age, exert their peculiar influence on health and mortality; and next to the effect of seasons, this influence of age calls for special consideration. Many statisticians of eminence, among others Villermé, Milne Edwards, Quetelet, and Lombard, have collected a large number of observations, which, although presenting many differences when taken individually, furnish on the whole tolerably uniform results. Accidental influences here disappear, and the operation of a natural law becomes apparent. The practical bearing and importance of this branch of statistical inquiry, must be obvious when we consider that the results refer directly to the proper method of preserving life in infancy and old age.

In order to throw light upon this subject, Dr. Casper has collected in an elaborate table no less than 40,000 deaths, which occurred at Berlin, during a period of seven years. It is derived from official documents in which the deaths were registered, according to months and periods of life. In the table, the deaths are arranged in seven sections; the first commencing with the deaths at and under one year of age, and the last including the deaths between the ages of 65 and 100. We here present a summary arranged according to the seasons.

| | 1 year. | 1-7 yrs. | 7-14. | 14-20. | 20-50. | 50-65. | 65-100, |
|---|---|---|---|---|---|---|---|
| Winter . . | 20·80 min. | 24·25 min. | 24·04 | 27·47 | 26·18 max. | 27·97 max. | 29·28 max. |
| Spring . . | 23·19 | 25·27 | 26·29 max. | 19·98 min. | 25·88 | 27·19 | 26·99 |
| Summer . . | 32·74 max. | 25·06 | 23·41 min. | 21·67 | 22·10 min. | 22·42 | 21·52 min. |
| Autumn . . | 23·21 | 25·34 max. | 26·20 | 30·83 max. | 25·66 | 22·37 min. | 22·15 |
| Difference between maximum and minimum. | 11·94 | 1·09 | 2·88 | 10·85 | 4·08 | 5·60 | 7·76 |

These numbers are calculated upon a basis of 100 deaths at the different ages corresponding to the different seasons. It will be apparent from this table, that the influence of the weather upon life, is very different at the different stages of existence. The greatest difference between the maximum and minimum of deaths exists during the age of infancy, and the least difference in the first septennial period. It shows further, how much may be accomplished by the adoption of proper precautions for the preservation of life. Children, under one year of age, are cut off in much greater proportion during the hot season of the year, i. e., in the months of July and August, than at other seasons; as for example, during winter, in which the deaths are actually fewest. No such disproportion in the number of deaths as here exists, is met with at any other period of life. Thus then it follows, that the heat of summer is more fatal to children than the cold of winter. This fact cannot be explained by the supposition that the number of births is greater during summer; for in Berlin, the maximum of births actually occurs in winter. While the researches of Schübler of Stuttgardt, and Emerson of Philadelphia, furnish similar results, those of Villermé in France, and Quetelet in Belgium, tend to show that the maximum mortality among infants occurs during winter, a conclusion which is in accordance with physiological principles, owing to the low state of the calorific function at this period of life. Dr. Casper refers this great mortality during winter, to the evil practice which exists in some Catholic countries, and in those localities where the provisions of the Code Napoleon are rigorously enforced, involving the exposure of new-born infants to the influence of cold, by carrying out the provisions for civil registration and by the adoption of early baptism. In other countries, where these practices are not adopted, the mortality of infancy is decidedly lower, because the child is withdrawn from the injurious effects of cold. The balance of mortality in other seasons, excepting the summer, differs but little from that of winter; the fact is—infants are very sensitive to *extremes* of temperature, and easily suffer from them. Hence the summer is so fatal to infant life; for it is far less easy to protect a child against the intense heat of summer, than against the severe cold of winter.

The second period of life, from one to seven years, is that in which the difference between the maximum mortality in autumn and the minimum in winter is the least,—reaching only to 1·09 per cent., from which we deduce the corollary, that the seasons exert the least influence at this period on life and death. This corresponds to what has been already announced by Dr. Lombard respecting the mortality of Geneva. From the age of two to fifteen years, he found that the seasons exerted scarcely any perceptible influence on the rate of mortality. In the second period of the Berlin table, the difference was only 2·88 per cent. between the maximum

(spring) and the minimum (summer); and this, therefore, is the most favourable age after the first septennial period. In the third septennial period, comprising the age of puberty (14—20), a difference exists almost as great as that which is found in early infancy. While about 20 per cent. at this age die in spring, more than 30 per cent. die in autumn. The tables of mortality throw no light upon the causes of this excessive difference produced by season. It is worthy of remark, however, that, from the twentieth year up to extreme old age, the winter is the most dangerous and the summer is the most favourable season for health; and it is curious to notice how gradually the maximum mortality for the winter season increases with the advance in years. As man progresses in life, the more necessary does warmth become to him;—while the summer lengthens, the winter shortens his days. This is already a fact well established by numerous researches;—those contained in the present paper only tend to confirm the conclusion at which writers on vital statistics have already arrived. According to Quetelet's tables of mortality in Belgium, the greatest number of deaths among individuals above twenty-five takes place in February, and the smallest number in July. While Schubler's researches in Stuttgardt show that, as in Berlin, the greatest number of children die in summer and the fewest in winter, the mortality is completely reversed as to adults—the greater number of these dying in the winter season. Lombard has proved, with respect to Geneva, that among adults the cold of winter regularly increases the amount of mortality, so that among individuals above eighty years of age, for one who dies in summer two die in winter. Buek states that in Hamburg the greater number of aged persons die in January, and of adults in February. Dr. Heberden remarked that the winter of 1795 was the coldest, and that of 1796 the hottest, until that time known in London; in the former thrice as many old persons died as in the latter. The important practical consequences respecting the regimenal and hygienic treatment of persons advanced in life are thus rendered sufficiently obvious; while they indicate the necessity, they confirm the utility of making the most extensive inquiries into the subject of vital statistics.

Dr. Casper has attached to this essay the following summary of practical conclusions:

1. In Berlin, while the month of January is the least, December is the most favourable to health.
2. The greatest number of deaths occur in spring, and the smallest number in summer.
3. Extremes of temperature are dangerous to life.
4. A high barometrical pressure tends to increase, while a low barometrical pressure tends to decrease, the rate of mortality.
5. The influence of atmospheric pressure on human life varies in different seasons.
6. No condition of the air is so dangerous to life as dry cold. On the contrary, humid cold has the greatest tendency to support life.
7. Of all seasons of the year, the winter gives rise to the greatest number of cases of inflammatory diseases, while the spring is most fatal to them, especially to cases of pneumonia.
8. Cold winters, warm springs, summers, and autumns increase the danger and fatality attendant on inflammation attacking the brain and respiratory organs; and *vice versâ*.

9. The maximum mortality from phthisis occurs in spring, and after this season in winter. The minimum mortality from this disease occurs in autumn and summer.

10. Variations in the state of the atmosphere appear to exert but little influence upon the relative number of deaths from phthisis.

11. Nervous fever is most frequent and fatal in autumn; it is least frequent and fatal in spring.

12. The influence of weather and season upon health varies with the different periods of life.

13. This influence is most marked in the ages of infancy and puberty, but it is least marked in the first septennial period of existence.

14. From the twentieth year upwards, the winter is the most dangerous, and the summer the most favorable season to life and health; and the older the individual the more striking is this difference.

The paper of which we next propose to give a brief analysis is one nearly allied to the foregoing, in which medical statistics are brought to bear upon a somewhat singular branch of inquiry, namely the *Influence of the periods of the day upon births and deaths.*

It is perhaps known to our readers, that some researches have been already made in respect to *births,* although without leading to any satisfactory results. The statistical tables of Ranken, Quetelet, and Buek, although differing from each other in data upon which the calculations are based, agree in representing that more children are born by night than by day. The most exact are those of Ranken, collected from 890 observations in his own midwifery practice, in which the hour of birth is uniformly stated. The tables of Quetelet include 2680 cases, observed in the Hospice de la Maternité, at Brussels, between the years 1811 and 1822. Buek's cases are not so satisfactory, because they have been loosely collected from various sources of private practice: hence but little reliance can be placed on them in reference to the question about to be discussed. Casper's data are derived from 809 cases occurring in the Maternity of Berlin, between the years 1830 and 1833. His results differ from those of Quetelet in the fact that they show a more gradual increase and decrease in the number of births at different periods. We cannot here find room for the number of cases which occurred at each hour, but the following table comprises the total cases which occurred to each observer in *terhoral* periods; and it shows not merely the absolute number of births, but also the relative proportion on a standard of 1000 cases:

| Hours of the Day. | Absolute Number of Births. | | | | | Relative Number of Births per 1000. | | | | |
|---|---|---|---|---|---|---|---|---|---|---|
| | Quetelet. | Buek. | Ranken. | Casper. | Totals. | Quetelet. | Buek. | Ranken. | Casper. | Totals. |
| 12-3 A.M. | 445 | 159 | 137 | 116 | 857 | 166 | 171 | 154 | 143 | 161 |
| 3-6 | 353 | 131 | 129 | 114 | 727 | 132 | 141 | 145 | 141 | 137 |
| 6-9 | 299 | 141 | 119 | 95 | 654 | 112 | 151 | 134 | 117 | 123 |
| 9-12 | 315 | 90 | 85 | 92 | 582 | 117 | 97 | 95 | 114 | 110 |
| 12-3 P.M. | 279 | 101 | 97 | 112 | 589 | 104 | 109 | 109 | 139 | 111 |
| 3-6 | 295 | 70 | 88 | 94 | 547 | 110 | 75 | 99 | 1[illegible]6 | 103 |
| 6-9 | 351 | 101 | 117 | 89 | 658 | 131 | 108 | 131 | 110 | 124 |
| 9-12 | 343 | 138 | 118 | 97 | 696 | 128 | 148 | 133 | 120 | 131 |
| | 2680 | 931 | 890 | 809 | 5310 | 1000 | 1000 | 1000 | 1000 | 1000 |

It will be seen from this extensive calculation of cases by various observers, that while the number of births between 9 o'clock in the evening and 6 o'clock in the morning exceeds the average (125), those between 9 o'clock in the morning and 6 o'clock in the evening are below the average. The greatest number of births takes place in the hours following midnight, and next to this, in the terhoral period between 3 and 6 o'clock in the morning. There is less agreement respecting the period at which the minimum of births occurs; but this Dr. Casper thinks may be owing to the unequal number of facts collected by the different observers. The author then proceeds to show that if the day be divided into sexhoral periods, the differences vanish, and the births then increase in frequency in the following proportions per 1000:

| | |
|---|---|
| From 12 to 6 A.M. | 296 |
| 6 to 12 | 232 |
| 12 to 6 P.M. | 215 |
| 6 to 12 | 257 |

Thus they occur most frequently in the hours after midnight; then in the hours before midnight; thirdly, in the hours before noon; and lastly, in the hours after noon. Dr. Casper refers to various hypotheses respecting the cause of this preponderance of births during the night, but none of them appear satisfactory. For the first time, he has endeavoured to determine whether the cause of the difference had any reference to the sex of the child, to its healthy or morbid development, to its maturity or immaturity, to its being a first, or a second, third, or fourth child,—to the delivery having been natural or instrumental. The result of his inquiries is, that none of these conditions have any demonstrable influence whatever on the period of the day at which the birth occurs.

The subject, however, has hitherto been regarded only in one point of view,—namely, the period at which the act of birth, i. e., the separation of the child from the mother, is completed. The time at which labour-pains first appear is obviously material to the inquiry; the exceptions here may have a smaller range than those which exist with regard to the delivery of the child, since this condition is more likely to be under the influence of a natural law. Besides Dr. Casper thinks it not improbable that some practical hint may be derived from an examination of this question, whereby the obstetric practitioner may be able to form a prognosis respecting the probable sex and the probable duration of labour. In illustration of these propositions, he presents us with a table of 787 births arranged according to sex, and according to whether the children were or were not those of primiparous females. We must, as usual, content ourselves with the results. The maximum number of cases in which labour pains come on, occurred in the terhoral period between *midnight and three o'clock in the morning;* the minimum number of cases were between *six and nine o'clock in the morning.* The numbers gradually approached each other between these two extremes. The proportion of cases in which labour pains come on at night, is much greater than that in which delivery occurs at night. Thus in the Berlin Maternity, for one day-birth there were 1·05 night-births; while the births in which the pains began by day were 1·31, those in which they began by night amounted to 1·75. It was also ascertained as a curious fact, that when the pains of labour began by day, the greatest number of male children were born; while when they commenced by night, there was a preponderance of female births.

The period at which labour pains came on appeared to have but a slight influence on the duration of labour. The average duration of a labour, from the number which occurred in the Berlin institution between the years 1830 and 1834, was about *nineteen hours*. In those births in which the pains commenced by day, the average duration was twenty hours; while in those in which they commenced by night it was eighteen hours. With respect to children born living and dead, the following facts were ascertained:

Out of 295 still-born children, the proportion in 1000, was for the four diurnal periods of six hours:

| | |
|---|---|
| From 12 to 6 A.M. | 275 |
| 6 to 12 | 230 |
| 12 to 6 P.M. | 193 |
| 6 to 12 | 302 |

The maximum, in regard to children born dead, unlike that of the general births, occurs in the six hours which precede midnight; but the minimum falls in both cases in the afternoon hours. The preponderance of night- over day-births, with respct to still-born children, is even greater than with respect to those born living.

*Influence of the periods of the day on mortality.* Proceeding on the principle that nothing connected with human life is accidental or dependent on chance, Dr. Casper next proceeds to examine the question respecting the relative rate of mortality at different hours of the day. This is just as much a legitimate object of medical inquiry as the determination of deaths at different seasons or in different months of the year. Our knowledge must, in all these cases, rest upon similar data, i. e., upon facts accurately observed and recorded. The researches hitherto made on this subject by Virey, Metzler, Quetelet, and Buek are, in the opinion of our author, insufficient to solve the question. The numbers are either too limited, or they have not been collected with sufficient attention to accuracy.

Casper's data consist of 5595 deaths, officially recorded in Berlin, by those who are appointed by government to verify death and report the cases. To make the results simple and intelligible, the number of deaths is taken at 100, and the day is divided into four sexhoral periods. It is obvious, if the rate of mortality was the same throughout the day, there would be 25 deaths in each period, but we find the following results:

| | |
|---|---|
| From 12 to 6 A.M. | 25 |
| to 12 | 29 |
| 1 to 6 P.M. | 25 |
| to 12 | 21 |

From this it will be perceived, that the maximum amount of mortality occurs in the six hours before noon, while the minimum rate occurs in the six hours after noon. The other two periods of the day appear to be indifferent. It is rather singular as a result, to find that the morning hours are those in which the greatest number of deaths occur; but it must not be forgotten that the period will be in many respects modified by the nature of the disease causing death. We consider it unnecessary to follow our author into his tables of mortality, as they are affected by the various classes of diseases. It may suffice to say, that they are of a most minute and extensive kind. We learn from them, as general facts, that *four fifths* of all deaths (5591 cases) are due to *chronic*, and *one fifth* to *acute diseases;* and, further, that the number of those deaths from acute diseases is smaller in the morning and larger in the afternoon hours, thus departing from the average results of the total deaths. The author attempts, but in our opinion not very satisfactorily, to account for this peculiarity in acute diseases. He then passes into an analysis of the differences observed in respect to the diurnal deaths for various classes of chronic disease, and discusses certain hypotheses respecting the causes of these differences. He afterwards examines the probable influence of seasons on the deaths at various diurnal periods. Our space is, however, already exhausted, and we shall therefore merely subjoin the general conclusions at which Dr. Casper has arrived respecting the statistics of births and deaths:

1. The greatest number of births occur between nine o'clock in the evening and six o'clock in the morning; while the smallest number occur between nine o'clock in the morning and six o'clock in the evening.

2. The pains of labour commence most frequently between twelve o'clock at night and three o'clock in the morning; least frequently from six to nine in the morning.

3. The influence of night is more marked with respect to the commencement of labour-pains than with respect to complete delivery.

4. Among those births in which the pains commenced by day, the greater number were male children, and *vice versâ.*

5. On an average, delivery was more protracted when the pains commenced by day than by night.

6. The preponderance of nocturnal over diurnal births is more striking in respect to children born dead, than in respect to those born living.

7. The maximum mortality occurs in the hours before noon, and the minimum mortality in the hours before midnight.

8. Individually regarded, the ratio of deaths from inflammations, phthisis, and pulmonary hemorrhage is greater in the afternoon; from fevers and exanthemata, just before midnight; from cerebral apoplexy, during the day; and from diseases of the nervous system in general in the hours which immediately follow midnight.

We here close for the present our examination of this volume. Our readers will perceive that we have given an analysis rather than a review of the principal medico-statistical papers which it contains. We have preferred in most cases allowing the author to speak for himself,—by no means pledging ourselves to the unqualified reception of the whole of his views. As we have before remarked, some difference of opinion will necessarily exist among professional men respecting the practical utility of statistical researches of this kind; but as the influence of weather and season upon health and mortality is a universally admitted truth, it is clear that the general influence can only be properly determined by minute researches like those of Professor Casper, on the special influences of heat and cold, high and low atmospheric pressure, dryness and humidity, which, in reality, constitute the complex elements of the weather, and on the various degrees of which, atmospheric changes depend. It would be difficult to point to any researches more accurate, more extensive, or more elaborate than those of the learned author of these essays, and there is no doubt, if ever it be permitted to the medical philosopher to determine the exact influence of the weather on the salubrity of places, and on the healthy condition of the human species, it will be by pursuing the course so well marked out by Casper. It would be absurd to pretend that such researches have yet led to anything like accuracy in prognosis; but in this respect this branch of medical statistics resembles many other parts of medical science. We cannot, however, fail to derive from such a condensation of well-observed facts new, more correct, and more enlarged views respecting the influence of atmospheric changes on health. There are many opinions on this subject which prevail extensively among the public as well as in the profession; some of them have arisen from inexact observations and hasty generalization, and yet they have been allowed to influence practice and hygienic treatment: others appear to have a better foundation in fact. If no other present benefit flow from these statistical researches, we have at least the satisfaction of knowing that they will enable us to distinguish the true from the false; and will teach us to be cautious in relying upon doctrines which have not been confirmed by experience and observation. We hope in the following number of this Journal to present an analysis of that portion of Dr. Casper's work which bears upon many novel and important questions in Legal Medicine.

---

